# MnmE, a Central tRNA-Modifying GTPase, Is Essential for the Growth, Pathogenicity, and Arginine Metabolism of *Streptococcus suis* Serotype 2

**DOI:** 10.3389/fcimb.2019.00173

**Published:** 2019-05-24

**Authors:** Ting Gao, Fangyan Yuan, Zewen Liu, Wei Liu, Danna Zhou, Keli Yang, Zhengying Duan, Rui Guo, Wan Liang, Qiao Hu, Yongxiang Tian, Rui Zhou

**Affiliations:** ^1^Key Laboratory of Prevention and Control Agents for Animal Bacteriosis (Ministry of Agriculture and Rural Affairs), Institute of Animal Husbandry and Veterinary, Hubei Academy of Agricultural Sciences, Wuhan, China; ^2^State Key Laboratory of Agricultural Microbiology, College of Veterinary Medicine, Huazhong Agricultural University, Wuhan, China; ^3^Cooperative Innovation Center of Sustainable Pig Production, Wuhan, China

**Keywords:** *Streptococcus suis* (*S. suis*), MnmE, tRNA modifying guanosine triphosphatase, tandem mass tag-based quantitative proteomics, growth, pathogenicity, arginine deiminase system

## Abstract

*Streptococcus suis* is an important pathogen in pigs and can also cause severe infections in humans. However, little is known about proteins associated with cell growth and pathogenicity of *S. suis*. In this study, a guanosine triphosphatase (GTPase) MnmE homolog was identified in a Chinese isolate (SC19) that drives a tRNA modification reaction. A *mnmE* deletion strain (Δ*mnmE*) and a complementation strain (CΔ*mnmE*) were constructed to systematically decode the characteristics and functions of MnmE both *in vitro* and *in vivo* studies via proteomic analysis. Phenotypic analysis revealed that the Δ*mnmE* strain displayed deficient growth, attenuated pathogenicity, and perturbation of the arginine metabolic pathway mediated by the arginine deiminase system (ADS). Consistently, tandem mass tag -based quantitative proteomics analysis confirmed that 365 proteins were differentially expressed (174 up- and 191 down-regulated) between strains Δ*mnmE* and SC19. Many proteins associated with DNA replication, cell division, and virulence were down-regulated. Particularly, the core enzymes of the ADS were significantly down-regulated in strain Δ*mnmE*. These data also provide putative molecular mechanisms for MnmE in cell growth and survival in an acidic environment. Therefore, we propose that MnmE, by its function as a central tRNA-modifying GTPase, is essential for cell growth, pathogenicity, as well as arginine metabolism of *S. suis*.

## Introduction

*Streptococcus suis* is an important zoonotic pathogen that causes a wide range of diseases, including meningitis, arthritis, pneumonia, and septicemia (Goyette-Desjardins et al., 2014). Human infection of *S. suis* is an emerging public health issue, while pig infection of *S. suis* causes severe economic losses worldwide (Feng et al., 2014). Two epidemic outbreaks of human *S. suis* serotype 2 (SS2) infection occurred in China in 1998 and 2005, which resulted in 229 infections and 52 deaths (Tang et al., 2006; Lun et al., 2007). Among the 33 serotypes classified on the basis of the antigenicity of capsular polysaccharides, SS2 is considered to be the most virulent and frequently encountered serotype isolated from clinically diseased pigs (Segura et al., 2017). More than 20 virulence-associated factors responsible for the pathogenicity of *S. suis* have been identified over the past four decades, which include suilysin, muramidase-released protein, two-component signal transduction systems, extracellular factors, fibronectin- and fibrinogen-binding proteins (FBPs), enolase, the arginine deiminase system (ADS), and glyceraldehyde-3-phosphate dehydrogenase (Jing et al., 2008; Feng et al., 2014; Fulde et al., 2014; Tan et al., 2017; Zhong et al., 2018).

*S. suis* naturally inhabits the upper respiratory tract, particularly the tonsils and nasal cavities, subsequently translocates across epithelial cell barriers, then reaches the bloodstream and disseminates through the circulatory system, and finally invades different host organs (Fittipaldi et al., 2012). Like other bacterial pathogens, *S. suis* may also encounter adverse situations during infection, such as oxidative stress, nutrient starvation, high osmotic pressure, and low pH. Thus, *S. suis* must adapt metabolically to survive *in vivo* and maintain pathogenesis (Willenborg et al., 2016). During this process, many proteins are either up- or down-regulated at the translation level in response to environmental stimuli and change (Gao et al., 2016). However, the underlying mechanisms of preferential regulation of proteins by *S. suis* during specific steps of host infection have not been clearly demonstrated.

tRNA modifications play important roles in the accuracy and efficiency of protein synthesis (Manickam et al., 2016). Changes in the modification levels at the wobble position of the codon affects the synthesis of specific proteins, which results in the adaptation of pleiotropic phenotypes through unknown mechanisms (Agris, 2008; Moukadiri et al., 2014). MnmE-like proteins, functioning as tRNA-modifying enzymes, are widely distributed in nature and conserved among Bacteria and Eukarya (Yim et al., 2006). MnmE, which is not only a tRNA-modifying enzyme, but also a guanosine triphosphatase (GTPase), together with GidA, forms an α2β2 heterotetrameric complex that controls the addition of a carboxymethyl aminomethyl (cmnm) group at position five of the wobble uridine of tRNA reading codons ending with an adenine or guanine (Shi et al., 2009; Fislage et al., 2014). Notably, this modification contributes a great deal to proper and efficient protein translation (Fislage et al., 2014). MnmE was first described in *Escherichia coli* and it has been found that null *mnmE* mutations are lethal in some *E*. *coli* strains, depending on the genetic background (Yim et al., 2003). Previous studies have also revealed that *mnmE* deletion leads to poor growth, especially at low temperatures, and MnmE of *Salmonella enterica* serovar Typhimurium and *Pseudomonas syringae* plays a potential role in pathogenic interactions with the host cell (Nilsson et al., 2017; Rodionova et al., 2018). Also, MnmE has been implicated in the bacterial response to stressors, other than low pH (Vivijs et al., 2016). Taken together, the above studies stress the importance of this protein in the mRNA decoding process. However, at present, little is known about the characterization and function of MnmE in *S. suis*.

The results of our previous study suggested a role for the tRNA-modifying enzyme GidA in the regulation of cell growth, capsule biosynthesis, and virulence of SS2 (Gao et al., 2016). The focus of the present study is MnmE, a second tRNA-modifying enzyme of SS2. Here, we show that the lack of *mnmE* induced poor growth, decreased pathogenicity, and perturbations to the arginine metabolic pathway. Tandem mass tag (TMT)-based quantitative proteomics was applied to investigate the biological characteristics and elucidate the regulatory mechanisms of MnmE in *S*. *suis*. Comparisons of the proteomic profiles identified differentially expressed proteins (DEPs) between the Δ*mnmE* strain and the wild-type strain SC19. Among 365 DEPs, 174 were down-regulated, and 191 up-regulated. Through analysis of these data at the system level, these DEPs were found to be mainly involved in cell division and growth, virulence, arginine biosynthesis, fatty acid biosynthesis, folate biosynthesis, nucleotide metabolism, and other cellular processes. Therefore, these data provide functional context that MnmE is crucial to cell growth, pathogenicity, and the arginine metabolic pathway of *S. suis*.

## Materials and Methods

### Ethics Statement

All experiments with the SS2 were performed in biosafety cabinets in biosecurity level 3 laboratory. All animal studies were conducted in strict accordance with the animal welfare guidelines of the World Organization for Animal Health. The animal study protocol was approved by the Ethics Committee of Huazhong Agricultural University (Wuhan, China) and conducted in accordance with the Hubei Province Laboratory Animal Management Regulations of 2005. All efforts were made to minimize animal suffering.

### Bacterial Strains, Plasmids, and Culture Conditions

The bacterial strains and plasmids used in this study are listed in Table 1. The virulent SS2 strain SC19 was isolated from a diseased pig during the 2005 epidemic outbreak in Sichuan of China (Li et al., 2009). The *S. suis* strains were grown in Todd-Hewitt broth (THB; Oxoid, Basingstoke, England) or on THB agar (THA; Oxoid, Basingstoke, England) plates supplemented with 5% sheep blood (Maojie, Nanjing, China) at 37°C. The arginine metabolic pathway study was performed using a chemically defined medium (van de Rijn and Kessler, 1980). Erythromycin (90 μg/mL) was added to screen for the mutant strain, while erythromycin (90 μg/mL), and spectinomycin (100 μg/mL) were added to select for the complemented strain. The *E. coli* DH5α strains were grown in LB broth (Difco Laboratories, Franklin Lakes, NJ, USA) or on LB agar plates at 37°C. If necessary, kanamycin (25 μg/mL) was added.

**Table 1:** Bacterial strains and plasmid used in this study.

**Strain or plasmid**	**Characteristics and function^**a**^**	**Source or reference**
**BACTERIAL STRAINS**		
SC19	*S. suis* serotype 2, the wide- type (Strep^r^)	(Li et al., 2009)
Δ*mnmE*	SC19 *mnmE*::*erm* (Strep^r^ Erm^r^)	This study
*E. coli* DH5α	Cloning host for recombinant vector	Trans
**PLASMID**		
pAT18	With an Erm^r^ gene expressing erythromycin resistance rRNA methylase	(Trieu-Cuot et al., 1991)
pET28a	Expression vector; Kan^r^	Novagen
pSET4s	*E. coli*—*S. suis* Shuttle vector; Spc^r^	(Takamatsu et al., 2001)
pSET4s-M	Derived from pSET4s for knocking out gene *mnmE* in SC19; Spc^r^ Erm^r^	This study
pSET2	*E. coli*—*S. suis* Shuttle vector; Spc^r^	(Takamatsu et al., 2001)
pSET2-CM	Derived from pSET2 for functional complementation of Δ*mnmE* (Spc^r^)	This study

### Construction of *mnmE* Gene Deletion and Complemented Strains

The *mnmE* deletion strain was obtained using an homologous recombination method (Takamatsu et al., 2001). Primers used in this study were designed according to the genome sequence of *S. suis* strain 05ZYH33 (GenBank accession number: CP000407) and are listed in Table 2. Primers Mup-F/Mup-R and Mdown-F/Mdown-R were used to amplify the upstream and downstream regions of *mnmE*. Moreover, the fragments were cloned into pSET4s, respectively. Finally, the *erm*^r^ expression cassette was amplified from pAT18 using the primers Erm-F/Erm-R and inserted between the upstream and downstream homologous arms of the recombinant pSET4s to achieve the *mnmE*-knockout vector pSET4s-M. The pSET4s-M was then electroporated into SC-19. The Δ*mnmE* mutant strain was screened on THB plates for sensitivity to spectinomycin and resistance to erythromycin. To confirm the mutant strain, the *mnmE* gene, the upstream, and downstream genes were amplified by PCR using the primer pairs *mnmE*-F/*mnmE*-R, 1453-F/1453-R, and 1455-F/1455-R, respectively. Moreover, Primers Test-F/Test-R were used to amplify the upstream to downstream regions of *mnmE* in SC19 and Δ*mnmE*, and the resulting DNA fragments were confirmed by DNA sequencing.

**Table 2:** Primers used for PCR amplification and detection.

**Primers**	**Primers sequence (5^**′**^-3^**′**^)**	**Amplification for**
Mup-F	AGGTCGACTCTAGAGGATCC TAATTTTTCTGGCGCATTCAGC	Upstream border of *mnmE*
Mup-R	CTCTTAAGTTTGCTTCTAAGAT ATCTTTCTTTCTAA	
	AATTGTCTTATCTATTAGTG	
Mdown-F	TACGGGGAATTTGTATCGA TGAACAAAAGTCATGT	Downstream border of *mnmE*
	AAGAAAACTTGC	
Mdown-R	AAACGACGGCCAGTGAAT TCTCTCTTGACCTGTTTG	
	GGTATTATAAA	
Erm-F	AATTTTAGAAAGAAAGATATC TTAGAAGCAAACTTA	Erm^r^ gene
	AGAGTGTGTTGA	
Erm-R	TTCTTACATGACTTTTGTTCATCGA TACAAATTCCCCGTAGG	
MnmE-F	CGGGATCCATGACACAC ACATTTGCAGA	*mnmE* gene
MnmE-R	CGCTCGAGTTAGTGACTGTCC TTTGATTT	
1453-F	ATGGTTGTATTTATCAAATC TAAAAAAGA	Upstream gene of *mnmE*
1453-R	TTAAGCTACAGCAAATAGCTTTTCTT	
1455-F	GTGAAAGGCGGCATTCCC	Downstream gene of *mnmE*
1455-R	TCAGTCATTTACCCACACCCC	
Test-F	GACAAAATAGCTGGTGTAATAAAG	Upstream to downstream regions of *mnmE*
Test-R	CTGTGTATGAAGGAGTTGAGGC	
CM-F	GATTCTGCAGTTTCGACGTCCTTTATC	*mnmE* gene and its
CM-R	CTCGGATCCTTATTTTCCAAGACAGA	Promoter

To construct a complemented strain, a DNA fragment containing the entire *mnmE* coding sequence and its promoter and terminator was amplified by using primers CM-F/CM-R. The promoter and coding sequences of the *mnmE* gene were amplified by PCR using the specific primer pair CM-F/CM-R. The amplicon was subsequently cloned into pSET2 to obtain the recombinant plasmid pSET2-CM, which was electroporated into strain Δ*mnmE*. The complemented strain CΔ*mnmE* was screened on THB plates for resistance to erythromycin and spectinomycin. To confirm the complemented strain, the *mnmE* gene, the upstream and downstream genes were amplified by PCR using three pairs of primers *mnmE*-F/*mnmE*-R, 1453-F/1453-R, and 1455-F/1455-R.

To further confirm the mutant strain Δ*mnmE* and the complementary strain CΔ*mnmE*, we also performed RT-PCR (Tan et al., 2015). Briefly, RNA was isolated using the Bacterial RNA Kit (Omega Bio-Tek, Inc., Norcross, GA, USA) in accordance with the manufacturer's instructions. In addition, cDNA was synthesized using the HiScript® II Q Select RT SuperMix for qPCR kit (Vazyme Biotech Co., Ltd., Nanjing, China) in accordance with the manufacturer's instructions. The primers *mnmE*-F/*mnmE*-R were used to confirm the deletion of *mnmE* gene, while, the primers 1453-F/1453-R (for upstream gene), and 1455-F/1455-R (for downstream gene) (Table 2) were used to confirm whether the upstream and downstream genes of *mnmE* were unaffected and functioning normally.

### Identification of Growth Characteristics

Growth rates of the SC19, Δ*mnmE*, and CΔ*mnmE* were detected through the measurement of the density changes represented by OD600 nm values and CFU counts of the cultures. Different strains were grown to the mid-exponential phase (OD_600_ of 0.6–0.8) in THB medium, then the cells were collected and washed in phosphate-buffered saline (PBS) three times, and the initial OD600 nm of all subcultures were adjusted to 0.07. Each of the subcultures was incubated at 37°C with rotating at 180 rpm and OD600 nm values were read every hour until the growth process entered the stationary phase. Meanwhile, a 100 μL aliquot of the bacterial culture was diluted and the number of viable bacteria was calculated every hour.

### Mouse Infection Experiments

To probe the possible role of the MnmE in *S. suis* virulence, 30 female specific-pathogen-free (SPF) Kun-Ming mice (6-week-old) weighing 26 to 30 g were randomly allocated to three groups (10 mice per group). Groups 1 and 2 were inoculated by intraperitoneal injection with 3 × 10^9^ CFU of either SC19 or Δ*mnmE*. Saline was administered to group 3 as a negative control. Clinical signs and survival time were recorded. The mice were observed for 7 days to obtain steady survival curves.

To better evaluate the pathogenicity of Δ*mnmE*, we performed a determination of viable bacteria in organs assay as described previously (Tan et al., 2017). Fifteen female SPF Kun-Ming mice (6-week-old) weighing 26 to 30 g were inoculated by intraperitoneal injection with 1 × 10^8^ CFU of a 1:1 mixture of mid-log-phase SC19 or Δ*mnmE*. Saline was applied to five mice as a negative control. At 12 h, 1 day, and 3 days post infection (dpi), brain, lung, spleen, and blood were obtained from five mice from each group. The samples were homogenized after weighing, and serial dilutions were plated onto THA. In order to count the colonies, we used 20 μg/mL streptomycin for SC19 and Δ*mnmE*, while 20 μg/mL streptomycin, and 90 μg/mL erythromycin were used for Δ*mnmE*.

### Adhesion and Invasion Assays

Adhesion and invasion assays were conducted in accordance with previously described methods (Ferrando et al., 2014). For the adhesion assay, HEp-2 cells were infected with mid-log-phase SS2 to reach a multiplicity of infection (MOI) of 100:1 (bacteria:cells) and then incubated for 30 min at 37°C. Unbound bacteria were removed by washing the cells gently with PBS three times and then cells were lysed in 1 mL of sterile distilled water. Adherent bacteria (cell-associated bacteria) were determined by plating a serial dilution of the lysates on THA plate. For the invasion assay, cells were incubated with bacteria for 2 h to allow for invasion and then incubated in medium containing penicillin (100 μg/mL) for 2 h to kill extracellular and surface-adherent bacteria. The numbers of invading bacteria were determined the same as the adhesion assay.

### Determination of Arginine Deiminase (AD) Activity

AD activity was determined by measuring the production of L-citrulline from L-arginine and evaluated according to the protocol described previously (Winterhoff et al., 2002; Xiong et al., 2014). Briefly, bacteria were grown in chemically defined medium to the mid-log phase and harvested by centrifugation. Then, cultures of test strains were lysed and, respectively, incubated for 2 h in 0.1 M potassium phosphate buffer containing 10 mM L-arginine at 37°C. Afterward, the enzymatic reaction was stopped by adding 250 μL of a 1:3 (v/v) mixture of 95% H_2_SO_4_ and 85% H_3_PO_4_, and 250 μL of 3% diacetylmonooxime solution were added to the samples incubating for 15 min at 100°C. Production of citrulline was determined colorimetrically at OD_450_. A citrulline standard and the uninoculated reagents were used as positive and blank controls, respectively. Results were expressed as nanomoles of citrulline produced per h per mg of whole cell protein.

### Determination of Ammonia in the Culture Supernatant

Ammonia production in the culture supernatant of wild-type, mutant, and complementary strains was quantified using an Ammonia Assay kit (Sigma-Aldrich Corporation, St. Louis, MO, USA) in accordance with the manufacturer's instructions.

### Protein Extraction, Digestion, and Labeling With TMT Reagents

SC19 and Δ*mnmE* cells at mid-log phase were cultured in THB as described above. Three independent biological replicates of bacterial pellets were then treated with SDT buffer (4% SDS,100 mM Tris-HCl, 1 mM DTT, pH 7.6) and heated for 15 min at 100°C. The cell suspensions were sonicated for 5 min (10 s of sonication with 15 s intervals) on ice and the protein concentration in the supernatants was determined using the Bradford protein assay. Each sample (200 μg) were digested with 3 μg of trypsin (Sigma-Aldrich Corporation) at 37°C for 16 h. The resulting tryptic peptides were labeled according to the protocol of TMT Reagent Kit (Thermo Fisher Scientific, Waltham, MA, USA). The labeled peptides were combined and fractionated by strong cation exchange (SCX) chromatography.

### Liquid Chromatography-Tandem Mass Spectrometry (LC-MS/MS) Analysis

After separation by SCX chromatography, equal amounts of digested protein were loaded into a Thermo Scientific EASY column (2 cm*100 μm 5 μm-C18) and then washed with solvent A (99% H2O, and 0.1% formic acid). By applying solvent B (84% acetonitrile, 16% H_2_O, and 0.1% formic acid), the peptides were eluted from the trapping column over an EASY-Column™ (75 μm × 100 mm, 3 μm, C18–42; Thermo Fisher Scientific) with a gradient (0–45% solvent B for 100 min, 35–100% solvent B for 8 min, 100% solvent B for 12 min at 300 nL/min) using the Easy nLC system (Thermo Fisher Scientific). For MS analysis, peptides were analyzed in positive ion mode. MS/MS was carried out with a Q-Exactive mass spectrometer (Thermo Finnigan LLC, San Jose, CA, USA) in the positive ion mode and a data-dependent manner choosing the most abundant precursor ions with a full MS scan from 300 to 1,800 m/z and resolution of 70,000 at m/z of 200. Target value determination was based on automatic gain control and dynamic exclusion duration was 60 s. MS/MS scans were acquired at a resolution of 35,000 at m/z of 200. Normalized collision energy was 30 eV and the underfill ratio was set at 0.1%.

### Proteomic Data Analysis

The data files produced by 15 fractions MS/MS were processed by Proteome Discoverer 1.4 and searched by Mascot 2.2 (Matrix Science, MA) against 83,725 *S. suis* protein-coding sequences deposited in the Uniprot database (downloaded on July 30, 2018). The search was conducted with trypsin applied as a specific enzyme and parameters used for normal peptides as follows: peptide mass tolerance, 20 ppm; fragment mass tolerance, 0.1 Da; max missed cleavages, 2; fixed modifications, carbamidomethyl (C), TMT 6/10plex (N-term), TMT 6/10plex (K); variable modifications: oxidation (M), TMT 6/10plex (Y); database pattern, decoy; and false-discovery rate ≤0.01 (Sandberg et al., 2012). Each of the identified proteins involved at least two unique peptides. Protein quantification was accomplished by correlating the relative intensities of reporter ions extracted from tandem mass spectra to that of the peptides selected for MS/MS fragmentation. To evaluate the DEPs between different strains, a fold change (Δ*mnmE*/SC19) > 1.2 and < 0.83, and a *p*-value of <0.05 were considered to represent up- or down-regulation, respectively. The mass spectrometry proteomics data have been deposited to the ProteomeXchange Consortium via the PRIDE partner repository with the dataset identifier PXD012716.

### Statistical Analysis

Statistical analyses were performed via unpaired Student's *t*-tests in GraphPad Prism 5 software (GraphPad Software, Inc., La Jolla, CA, USA) all experiments were performed in triplicate at least three times. All data are expressed as the mean ± the standard error of the mean. A *p*-value of <0.05 was considered as the threshold for significance.

## Results

### Construction and Confirmation of Δ*mnmE* and CΔ*mnmE*

**Figure 1:**
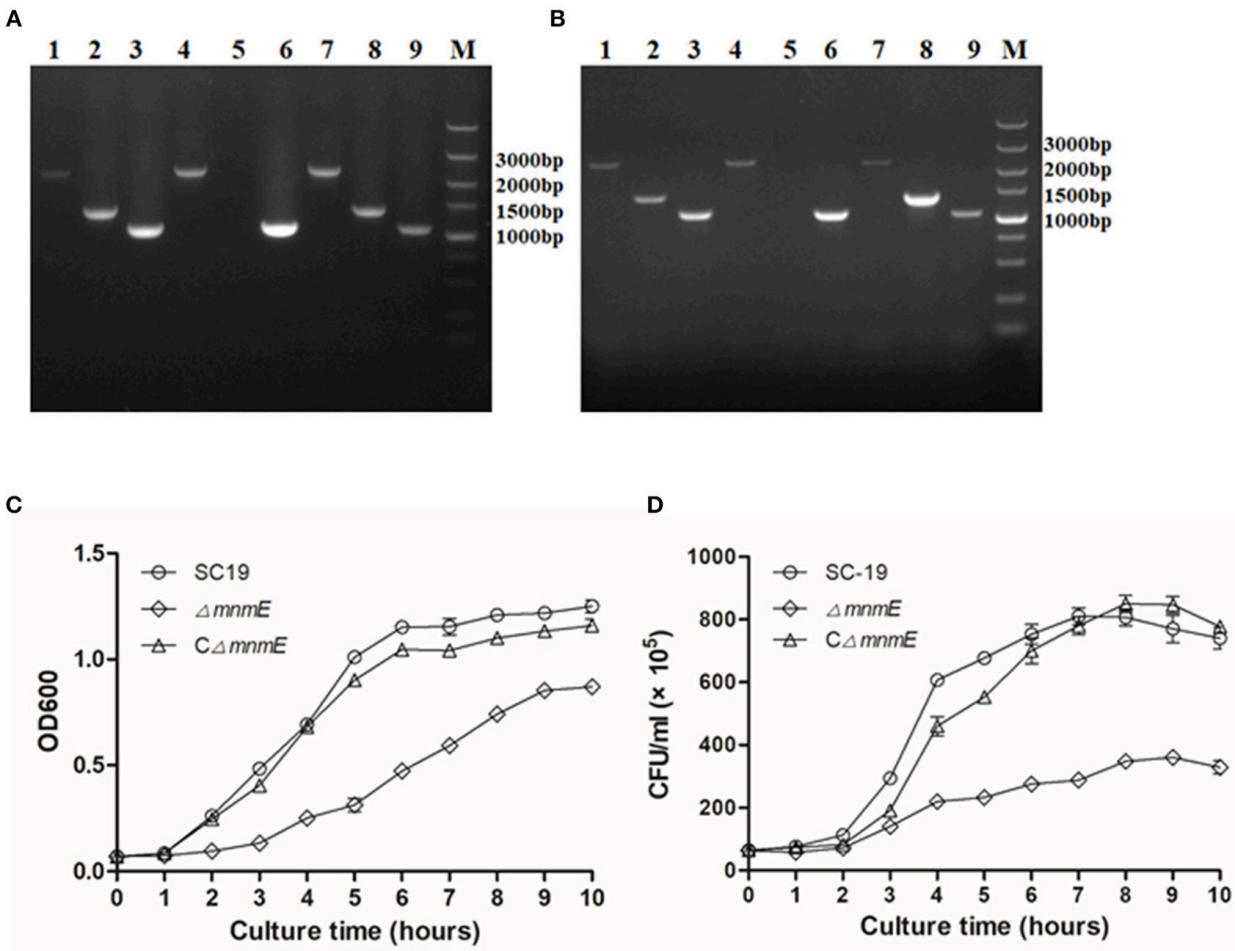
Confirmation and Characterization of the isogenic mutant Δ*mnmE*. **(A)** Combined PCR analyses of the Δ*mnmE* mutant. Lanes 1 to 3 represent the amplification of the upstream gene, *mnmE* gene, and downstream gene of SC19 using the primer set *mnmE*-F/*mnmE*-R, 1453-F/1453-R, and 1455-F/1455-R. Lanes 4 to 6 represent the amplification of the upstream gene, *mnmE* gene and downstream gene of Δ*mnmE* using the primer set *mnmE*-F/*mnmE*-R, 1453-F/1453-R, and 1455-F/1455-R. Lanes 7 to 9 represent the amplification of the upstream gene, *mnmE* gene and downstream gene of CΔ*mnmE* using the primer set *mnmE*-F/*mnmE*-R, 1453-F/1453-R, and 1455-F/1455-R. **(B)** Confirmation of the Δ*mnmE* mutant by RT-PCR. Lanes 1, 4, and 7 represent the amplification of upstream gene of *mnmE* using the primer set 1453-F/1453-R. Lanes 2, 5, and 8 represent the amplification of *mnmE* using primer set mnmE-F/mnmE-R. Lanes 3, 6, and 9 represent the amplification of downstream gene of *mnmE* using the primer set 1455-F/1455-R. Lanes 1, 2, and 3 use cDNA of SC19 as templates, whereas Lanes 4, 5, and 6 use cDNA of Δ*mnmE* as templates, Lanes 7, 8, and 9 use cDNA of CΔ*mnmE* as templates. **(C)** Growth curves of the strains. Bacterial cell density was measured spectrometrically at 600 nm. Data were collected at the indicated times. **(D)** CFU count of the strains. Separate aliquots of the bacterial suspensions were serially diluted and plated to determine CFU numbers per milliliter. Data were collected at the indicated times.

Colonies sensitive to spectinomycin and resistant to erythromycin were selected as candidates of *mnmE*-deletion mutants, and confirmed by PCR and RT-PCR (Figures 1A,B). At the gene and transcript levels of *mnmE* was expressed in strains SC19 and CΔ*mnmE*, but not in strain Δ*mnmE*. RT-PCR analysis also showed that the transcripts of the genes upstream and downstream from *mnmE* were not affected by the *mnmE* deletion, which could exclude associated polarity effects.

### Growth Phenotype of the Δ*mnmE* Mutant

First, the growth kinetics of the mutant strain were characterized. The SC19, Δ*mnmE*, and CΔ*mnmE* strains were grown to the mid-log phase and then inoculated in fresh THB at 37°C while rotating at 180 rpm. The growth rate of the *mnmE*-deficient cells was reduced as compared to that of strain SC19. Meanwhile, the CFU counts also showed that strain Δ*mnmE* grew much slower than strain SC19 (Figures 1C,D). Indeed, complementation of *mnmE* (CΔ*mnmE*) almost restored the growth defect. Notably, the colonies of Δ*mnmE* appeared smaller than those of SC19 when cultured on THB plates overnight. Based on these observations, MnmE appears to be critical for *S. suis* growth.

### Attenuated Pathogenicity in Mice

**Figure 2:**
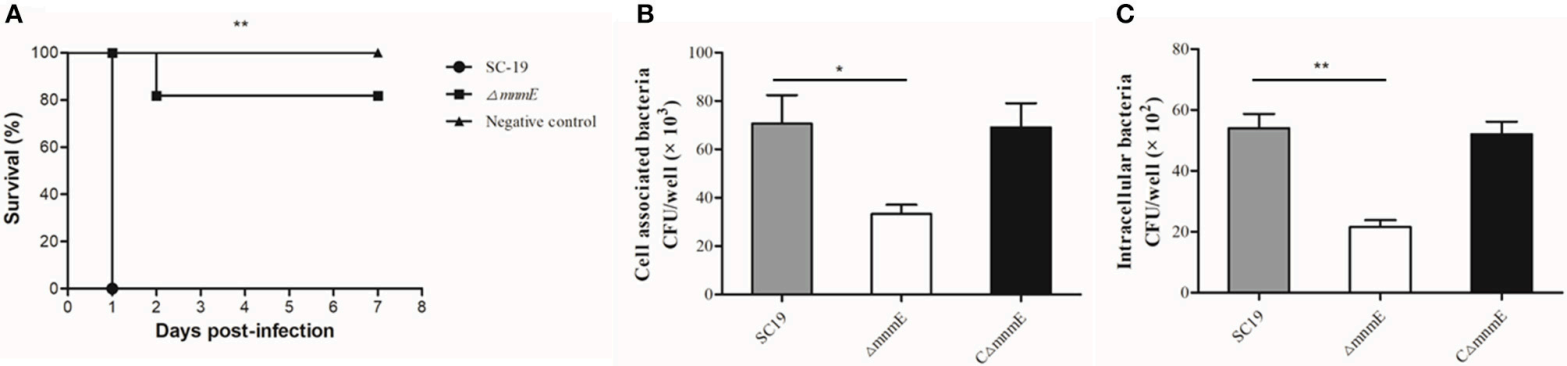
MnmE contributes to SS2 pathogenicity *in vivo* and *in vitro*. **(A)** Survival curves for mice in experiment infection. Ten mice in each group were separately injected intraperitoneally with 3 × 10^9^ CFU/mice of SC19 and Δ*mnmE*. Ten mice were inoculated with saline and served as negative control. Significant difference in survival between different groups were analyzed by Log Rank test (*p* < 0.01). **(B)** Cell-associated bacteria recovered after incubation with HEp-2 cells. The mutant strain Δ*mnmE* showed significantly reduced levels of adherence to HEp-2 cells compared with the degree of adherence of SC19 and CΔ*mnmE* (*p* < 0.05). **(C)** Bacteria invasion of HEp-2 cells. The mutant strain Δ*mnmE* showed significantly reduced levels of invasion of HEp-2 cells compared with that of SC19 and CΔ*mnmE* (*p* < 0.01). Statistical significance was determined by two-tailed *t*-test (^*^p < 0.05; ^**^p < 0.01).

Mice were experimentally infected to estimate differences in viability between strains Δ*mnmE* and SC19 *in vivo*. All 10 SC19-infected mice developed severe clinical symptoms, such as septicemia and meningitis, and died within the first day of infection. By contrast, the symptoms of the Δ*mnmE*-infected mice were milder and the mortality rate was relatively low (2/10) during the 7-day observational period (Figure 2A), indicating that the pathogenicity of Δ*mnmE* was markedly attenuated.

**Figure 3:**
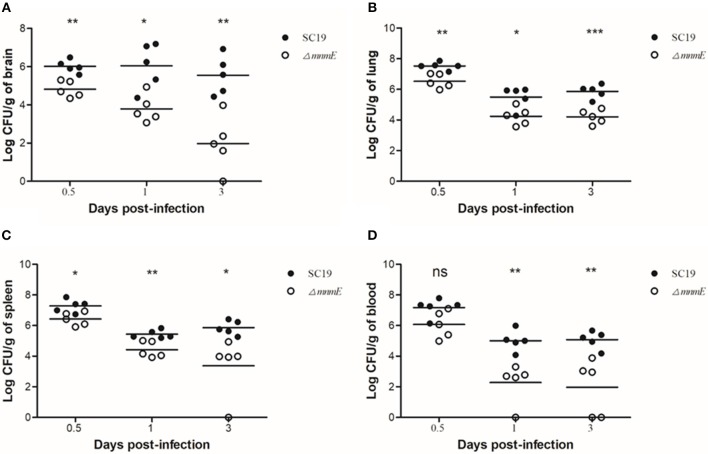
Bacteria loads in different mouse organs. Bacteria loads in **(A)** brain, **(B)** lung, **(C)** in spleen, and **(D)** in blood. Mice were inoculated intraperitoneally with 1 × 10^8^ CFU of a 1:1 mixture of mid-log phase SC19 and Δ*mnmE*. The survival strains were enumerated by plating serial dilutions of the samples on selective plates. Data are the result of CFU/g or CFU/ml in different organs analyzed per sample ± SEM. Statistical significance was determined by two-tailed *t*-test (ns, *p* > 0.05; ^*^p < 0.05; ^**^p < 0.01; ^***^p < 0.001).

To further evaluate the pathogenicity of Δ*mnmE*, a colonization experiment was performed using the intraperitoneal route of inoculation. First, the optical density at 600 nm (OD_600_) and CFU counts showed that *in vitro*, strain Δ*mnmE* grew much slower than strain SC19, and the colonies of Δ*mnmE* appeared smaller as compared to strain SC19. Hence, we investigated whether the characteristics would be the same under *in vivo* conditions. As expected, the *in vivo* adaptability of Δ*mnmE* was decreased as compared to that of SC19. Bacteria were recovered from brain, lung, spleen, and blood samples at different time points post infection. The bacterial loads of Δ*mnmE* in these tissues were lower than those of SC19 from 12 h to 3 dpi, and the mutant strain was partly cleared at 3 dpi (Figures 3A–D).

### Impaired Abilities of Adhesion to and Invasion in Epithelial Cells

Next, the role of *mnmE* in the adhesion to and invasion in host cells was investigated. The efficiencies of SC19 and its derivatives to adhere to and invade in HEp-2 cells were calculated. The binding and invasion rates of SC19 to the HEp-2 cells were 2- and 2.5-fold greater than that of Δ*mnmE* (Figures 2B,C), indicating that the inactivation of *mnmE* impaired the capacity of *S*. *suis* to adhere to and invade in epithelial cells.

### ADS-Related Metabolism

#### Reduced AD Activity

**Figure 4:**
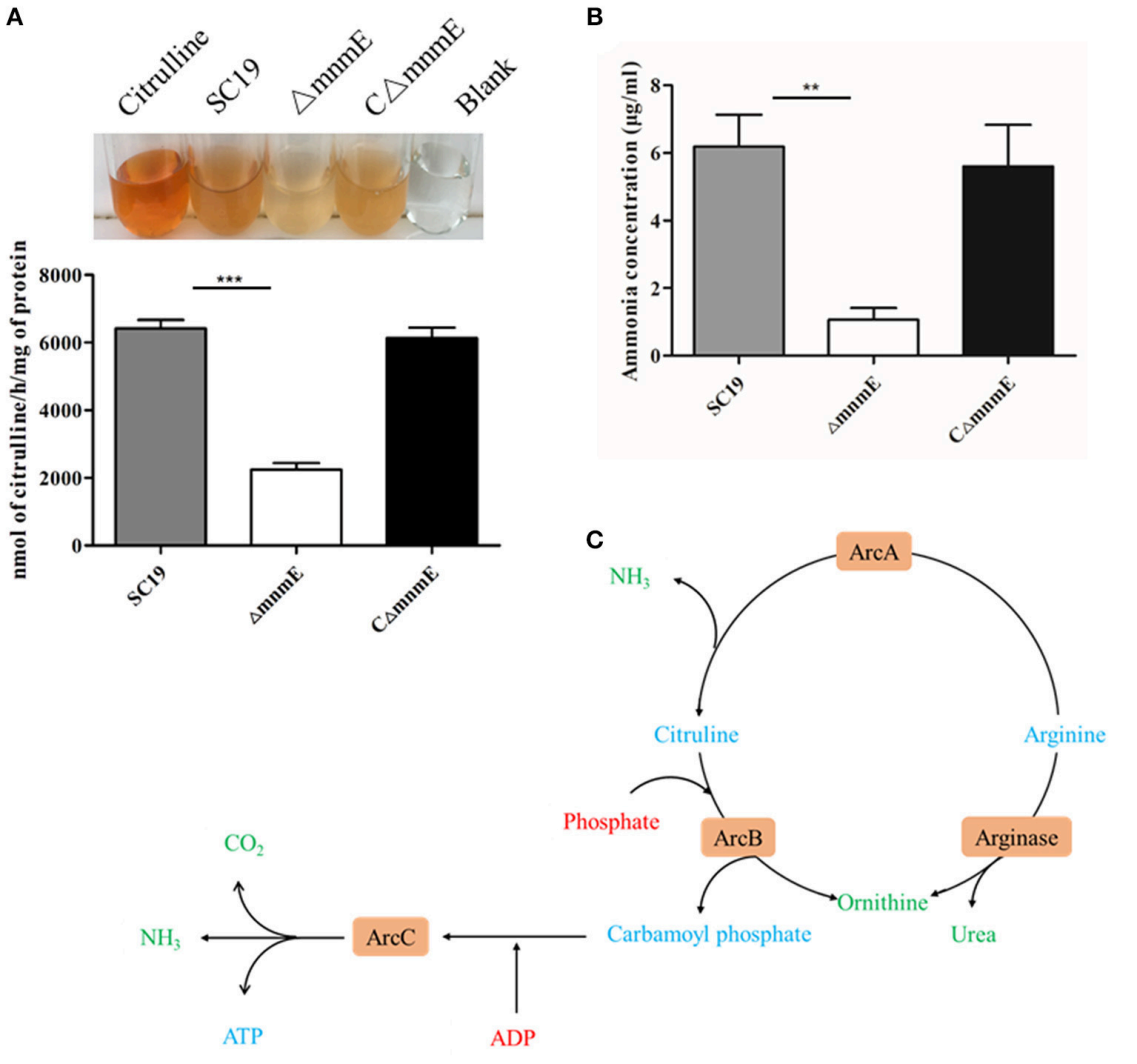
Regulation of the ADS by MnmE. **(A)** AD activities of different *S. suis*. Results were expressed as nanomoles of citrulline produced per hour per milligram of whole cell protein. **(B)** Production of ammonia in different *S. suis*. Ammonia production in supernatant of different *S. suis* is given as μg ammonia / ml. Data are presented as the mean ± SEM of a representative experiment performed intriplicate. Statistical significance was determined by two-tailed *t*-test (^**^p < 0.01; ^***^p < 0.001). **(C)** Schematic representation of *S. suis* arginine metabolic pathways. The core ADS enzymes (ArcA, ArcB, ArcC) facilitating the conversion from arginine to ornithine are depicted in orange. Metabolic intermediates are indicated in blue. The input of energy in terms of phosphate or phosphate derivatives (ADP) are marked in red, non-catabolized, and excreted products have a green color.

The results of a qualitative assay of the ADS showed that, similar to the positive control (citrulline standard), cellular extracts prepared from strains SC19, Δ*mnmE*, and CΔ*mnmE*, generated different degrees of orange color (Figure 4A), thereby confirming citrulline production. Cell extracts from both the wild-type and complemented strains yielded a darker orange color, as compared to the mutant strain. These results demonstrate that the deletion of *mnmE* down-regulated activities of the ADS. Therefore, we hypothesized that MnmE might affect *S. suis* arginine deiminase expression, in addition to its traditional role as a GTPase.

#### Lowered Ammonia Production

To further investigate the regulatory role of MnmE on arginine metabolism, ammonia production in the supernatant was determined for the wild-type, mutant, and complemented strains. After overnight incubation, the amount of ammonia in the mutant culture supernatant was significantly reduced by 5.8-fold as compared with the wild-type (Figure 4B), indicating that MnmE is required for adequate function of the ADS of *S*. *suis*.

### TMT-Based Quantitative Proteomics Revealed Different Expression Profiles Between Strains SC19 and Δ*mnmE*

To understand the underlying molecular mechanisms of the phenotype of Δ*mnmE*, TMT-based MS profiles of proteins produced by strains SC19 and Δ*mnmE* were applied. In this study, a total of 1,619 proteins were identified and quantified via TMT proteomics, of which 365 were DEPs, including 174 that were up-regulated and 191 down-regulated (Table S1).

**Figure 5:**
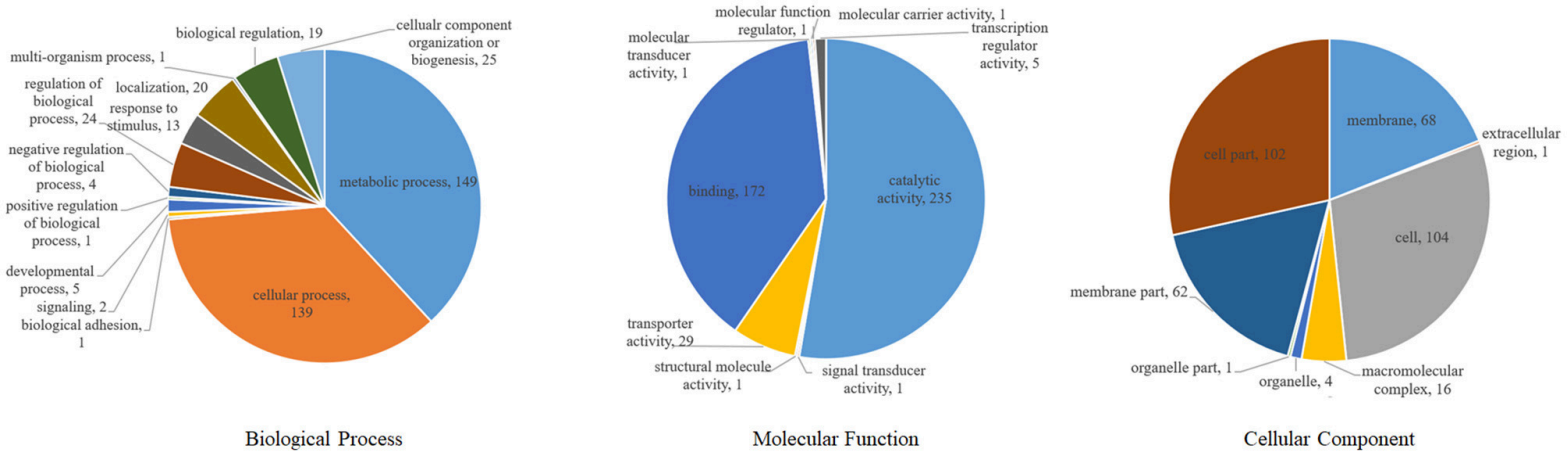
Classification of differentially expressed proteins in *S. suis* according to GO annotation.

According to gene ontology analysis, the 365 DEPs were classified into biological processes, cellular components, and molecular functions (Figure 5). The most prevalent biological process were metabolic processes (149, 40.8%) and cellular processes (139, 38.1%), which are the most important responses of *S. suis* to environmental stressors. The top two molecular functions were catalytic activity (235, 64.4%) and binding (122, 47.1%). The most common cellular components were the cell (104, 28.5%), followed by cell parts (102, 27.9%), the membrane (68, 19.1%), and membrane parts (62, 17.0%).

#### Proteins Associated With Cell Growth and Division

The *mnmE* deletion significantly reduced the growth rate of *S. suis*. Many proteins associated with cell growth and division were found to be regulated in the mutant strain (Table 3). These DEPs were mainly assigned into three classes: (i) eight proteins involved in DNA replication, recombination, and repair, which were all down-regulated, including DnaE (SSU05_0542), TopA (SSU05_0985), and XerD (SSU05_1702); (ii) three involved in cell division, including two that were down-regulated, i.e., STK (SSU05_0428) and FtsK (SSU05_1335); and (iii) five involved in cell wall biosynthesis, including four that were down-regulated proteins, i.e., MurD (SSU05_0476), MurE (SSU05_0651), MurG (SSU05_0477), and Pbp1A (SSU05_0414).

**Table 3:** Differentially expressed proteins associated with cell growth and division, virulence, and fatty acid metabolism.

**Protein name**	**Locus**	**Functions**	**Ratio(Δ*mnmE*/SC19)**	**Unique peptides**	**Sequence coverage(%)**
**CELL GROWTH AND DIVISION**					
XerD	SSU05_1702	Site-specific tyrosine recombinase XerD-like protein	0.5665	2	7.82
STK	SSU05_0428	Serine/threonine protein kinase	0.7039	20	35.54
YjqA	SSU05_1576	Superfamily I DNA/RNA helicase	0.6447	4	27.56
PcrA	SSU05_0731	Superfamily I DNA and RNA helicase	0.6687	15	18.45
PrpC	SSU05_0472	Protein phosphatase	0.6770	11	59.59
DnaE	SSU05_0542	DNA-directed DNA polymerase	0.6776	20	20.66
TopA	SSU05_0985	DNA topoisomerase I	0.7188	23	35.29
MurG	SSU05_0477	UDP-N-acetylglucosamine	0.7245	7	28.81
MurD	SSU05_0476	D-glutamic acid-adding enzyme	0.7625	19	55.68
FtsK	SSU05_1335	DNA segregation ATPase	0.7736	23	27.50
MutS	SSU05_2123	DNA mismatch repair protein	0.8078	17	22.10
Pbp1A	SSU05_0414	Penicillin-binding protein	0.8134	24	36.88
MurE	SSU05_0651	UDP-MurNAc-tripeptide synthetase	0.8158	11	27.70
ParB	SSU05_2193	Chromosome partitioning protein	1.2174	6	22.44
MurB	SSU05_0804	UDP-N-acetylmuramate dehydrogenase	1.2363	10	37.09
FtsE	SSU05_1411	Cell division ATP-binding protein	1.2679	12	51.74
**VIRULENCE-ASSOCIATED PROTEINS**					
SadP	SSU05_0272	Translation initiation factor 2 GTPase	0.5742	16	47.28
STK	SSU05_0428	Serine/threonine protein kinase	0.7039	20	35.54
ArcA	SSU05_0624	Arginine deiminase	0.3873	21	56.48
ArcB	SSU05_0626	Ornithine carbamoyltransferase	0.5153	13	45.99
ArcC	SSU05_0627	Carbamate kinase	0.3393	2	6.67
Sao	SSU05_1371	Surface antigen	0.6640	28	64.33
FBPS	SSU05_1942	Fibronectin/fibrinogen binding protein	0.7879	15	30.07
ZnuA	SSU05_2086	Adenylosuccinate synthase	0.8299	2	50.47
**FATTY ACID BIOSYNTHESIS**					
AccA	SSU05_1796	Acetyl-coenzyme A carboxylase carboxyl transferase subunit alpha	0.2905	6	19.46
AccD	SSU05_1797	Acetyl-coenzyme A carboxylase carboxyl transferase subunit beta	0.2444	8	31.25
AccC	SSU05_1799	Biotin carboxylase	0.3680	10	28.88
FabZ	SSU05_1800	3-hydroxyacyl-[acyl-carrier-protein] dehydratase	0.3717	2	17.14
FabF	SSU05_1802	3-oxoacyl-[acyl-carrier-protein] synthase 2	0.18734	8	26.52
FabG1	SSU05_1803	3-ketoacyl-ACP reductase	0.2132	13	80.33
FabD	SSU05_1804	Malonyl CoA-acyl carrier protein transacylase	0.4977	6	23.86
FabI	SSU05_1805	2-nitropropane dioxygenase	0.2033	2	39.56
FadB	SSU05_1809	Enoyl-CoA hydratase	0.4785	9	34.22

#### Virulence-Associated Proteins

Strikingly, the *mnmE* deletion reduced the virulence of *S. suis*. Several virulence factors were down-regulated in the mutant strain (Table 3). These DEPs were mainly assigned into two classes: (i) two proteins, i.e., FbpS (SSU05_1942) and Sao (SSU05_1371), involved in surface/secreted components, which were both down-regulated; and (ii) six (i.e., ArcA, ArcB, ArcC, STK, SadP, and ZnuA) involved in enzyme complexes, which were all down-regulated.

#### Pathway Perturbation by mnmE

To obtain more functional information of the DEPs between strains SC19 and Δ*mnmE*, Kyoto Encyclopedia of Genes and Genomes (KEGG) pathway (*p* < 0.05) enrichment analysis was conducted. Seven metabolic pathways were significantly perturbed by the deletion of *mnmE* (Table 4). Among these, four pathways, namely, lipid metabolism (ko00061), amino acid metabolism (ko00290 and ko00220), nucleotide metabolism (ko00230), as well as one as a cofactor and two that have roles in the vitamin metabolic pathway (ko00770, ko00780, and ko00790, respectively) are crucial for the central metabolism of bacterial cells, as well as bacterial growth and virulence. All proteins in the fatty acid biosynthesis pathway were significantly down-regulated (Table S1), which included AccA (SSU05_1796), AccC (SSU05_1799), AccD (SSU05_1797), FabZ (SSU05_1800), FabF (SSU05_1802), and FabD (SSU05_1804). Notably, the expression levels of the proteins ArcA (SSU05_0624), ArcB (SSU05_0626), ArcC (SSU05_0627) in the arginine biosynthesis pathway were decreased in the Δ*mnmE* mutant (Table 3). In addition, 14 (70%) of 20 proteins involved in purine metabolism were down-regulated (Table S1).

**Table 4:** Perturbed pathways in the Δ*mnmE* mutant strain.

**Pathway**	***p*-value**	**richFactor**	**Pathway ID**
**LIPID METABOLISM**			
Fatty acid biosynthesis	0.000096	0.80	ko00061
**AMINO ACID METABOLISM**			
Valine, leucine, and isoleucine biosynthesis	0.000450	0.89	ko00290
Arginine biosynthesis	0.011367	0.83	ko00220
**NUCLEOTIDE METABOLISM**			
Purine metabolism	0.006480	0.46	ko00230
**COFACTORS AND VITAMINS METABOLISM**			
Folate biosynthesis	0.011776	0.75	ko00790
Biotin metabolism	0.027770	1	ko00780
Pantothenate and CoA biosynthesis	0.040289	0.58	ko00770

## Discussion

MnmE is an evolutionarily conserved tRNA-modifying enzyme that contributes to correct interactions between codons and anticodons during translation in both eukaryotic and prokaryotic cells (Fislage et al., 2014). MSS1 is a homolog of the *mnmE* genes of both humans and yeast (Decoster et al., 1993). The protein product of MSS1 from yeast is localized in the mitochondria and mutants of these proteins are associated with severe respiratory defects (Umeda et al., 2005). Meanwhile, in *E*. *coli*, MnmE is relevant to cell growth, and mutations are lethal in specific strains (Martinez-Vicente et al., 2005). Also, in *S. enterica* serovar Typhimurium, MnmE is involved in bacterial growth and virulence (Shippy and Fadl, 2014; Nilsson et al., 2017). *S. suis* is an important emerging zoonotic pathogen, however, the role of MnmE in *S. suis* remains unclear. As a tRNA-modifying enzyme, MnmE affects protein translation efficiency and fidelity. Therefore, in the present study, TMT-based quantitative proteomics were applied to identify proteins and metabolic pathways regulated by MnmE. The results showed that *S. suis* MnmE can regulate the expression of proteins involved in bacterial central metabolism and virulence, thereby explaining the attenuated growth, pathogenicity, and perturbations to the ADS metabolic pathway of the *mnmE* knock-out strain.

### MnmE Is Essential for *S. suis* Cell Growth

Inactivation of *S. suis* MnmE affects the bacterial phenotype, including the size of the colonies. There are two reasons for this phenotype: (i) reduced growth, as verified by CFU counts and absorbance at OD_600_; and (ii) the smaller size of strain Δ*mnmE*, as compared to strain SC19. Analysis of cell growth kinetics and transmission electron microscopy were both performed in this study. CFU counts and OD_600_ readings revealed that strain Δ*mnmE* grew much slower than strain SC19 and there was no obvious difference in the cell size between strains, as observed via transmission electron microscopy (Figure S1).

These results indicate that MnmE is involved in the growth regulation of *S. suis*. This finding is in agreement with those of previous reports on *E. coli* and *S. enterica* serovar Typhimurium. To further understand the molecular mechanism of MnmE on growth regulation, TMT-based proteomic analysis was conducted. According to the DPE analysis, many proteins involved in cell growth and division were down-regulated in the Δ*mnmE* mutant, which included three classes of proteins: (i) those related to DNA replication, recombination, and repair, such as DNA/RNA helicases (YjqA and PcrA), DNA topoisomerase I (TopA), DNA/RNA helicase (PcrA), site-specific recombinase (XerD), DNA mismatch repair protein (MutS), DNA-directed DNA polymerase (DnaE), and ribonuclease (RNY); (ii) cell division, including FtsK and StpK; and (iii) cell wall biosynthesis, including MurD, MurE, MurG, and PBP1A, which positively regulate cell division (Eniyan et al., 2018; Szafran et al., 2018; Zucchini et al., 2018). We previously reported that STK, a serine/threonine protein kinase, regulates cell growth and division. Phosphoproteomics revealed that the down-regulated substrates of STK were all crucial cell division-associated proteins, which included FtsA, GpsB, DivIVA, and MapZ (Zhang et al., 2017). Therefore, down-regulation of STK could contribute to the slow growth rate of the Δ*mnmE* mutant. As we know, cell wall peptidoglycan (PG) is vital for bacterial survival and normal cell growth, and thus is an attractive drug target in bacterial infection (Bhat et al., 2017). The cytoplasmic biosynthesis of PG is a complex process that involves six MurA-F enzymatic reactions to catalyze the formation of PG units from the primary substrate uridine diphosphate N-acetylglucosamine (Eniyan et al., 2018). In addition, PBP1A, which catalyzes the cross-linking reaction of polymeric glycan chains (Frere and Page, 2014), was also down-regulated. However, two cell growth and division-associated proteins, MurB and FtsE, were up-regulated. The MurB enzyme belongs to a family of proteins that contain FAD-binding domains and share a characteristic FAD binding fold (Eniyan et al., 2018), the same as GidA, which is the partner of MnmE. As mentioned above, GidA and MnmE form an α2β2 heterotetrameric complex that controls the addition of a cmnm group at the wobble position of tRNA molecules (Moukadiri et al., 2014). Based on these considerations, we suppose that the deletion of *mnmE* renders GidA more available for FAD-binding. Thus, the mutant up-regulates MurB to compete with GidA for FAD-binding. FtsE together with FtsX forms a dimer that acts as an ABC transporter. The FtsEX protein complex plays a major role in the regulation of PG hydrolases in response to signals from cell division (Sham et al., 2013). Any disruption, either through inhibition or overactivation of the hydrolases, results in the inability to maintain the function of the cell wall (Margulieux et al., 2017), therefore, up-regulation of FtsE may impair cell growth.

### MnmE Is Essential for *S. suis* Pathogenicity

The deletion of *mnmE* in *S. suis* also resulted in an alteration to bacterial pathogenicity. *In vivo* and *ex vivo* studies revealed that Δ*mnmE* is associated with decreased mortality (Figure 2A) and bacterial loads in mice (Figure 3), as well as reduced adhesion to and invasion in epithelial cells (Figures 2B,C). This attenuation may result from the impaired growth of Δ*mnmE* or impaired translation efficiency of the virulence-associated proteins.

In this study, analysis of protein expression profiles indicated that several proteins associated with adherence and hemolysis were repressed in Δ*mnmE*, such as FBPs and SadP (Ge et al., 2004). A considerable number of FBPs of various bacterial species are crucial virulence factors (Joh et al., 1999). In *S. pyogenes*, FBPs were expressed in the human host and preferentially mediated adherence to human buccal epithelial cells (Courtney et al., 1996). In *S. suis*, FBPs play roles in the colonization of specific organs during infection (de Greeff, 2002). Our results showed that *mnmE* disruption decreased the abilities of *S*. *suis* to adhere to and invade in epithelial cells, which can be explained by the down-regulation of the above DEPs. As previously reported, the tRNA-modifying GTPase MnmE has been implicated in the bacterial response to stressors other than low pH. In this study, we found that the ADS of *S*. *suis* (ArcABC), which was down-regulated, is involved in the adhesion to and invasion in epithelial cells (Degnan et al., 2000; Fulde et al., 2014), as well as resistance to oxygen, nutrient starvation, and acidic environments (Gruening et al., 2006). Here, only the arginine repressor ArgR was upregulated. ArgR is an essential transcriptional regulator that specifically regulates the arcABC operon by directly interacting with its promoter (Fulde et al., 2011). Interestingly, the protein expression of ArcABC was remarkably down-regulated rather than up-regulated, indicating that the translation efficiency mediated by MnmE has a major impact on ADS expression far beyond the role of ArgR in transcriptional activation. The biological significance of tRNA modification by MnmE on these virulence-associated proteins warrants further investigations.

### MnmE Is Essential for *S. suis* Metabolism

According to the KEGG pathway analysis results, seven metabolic pathways were significantly perturbed following *mnmE* deletion (Table 4). The fatty acid biosynthesis, arginine biosynthesis, and purine metabolic pathways were inhibited, while the valine, leucine, and isoleucine biosynthesis, cofactor, and vitamin metabolic pathways were activated. These perturbations could be related to the impaired growth and virulence of Δ*mnmE*.

Lipid metabolism is thought to play a key role in the pathogenicity of several bacteria (Bohl et al., 2018; Ghazaei, 2018; Rameshwaram et al., 2018). There are three main functions provided by this pathway in bacteria: (i) the activation of bacterial lipolytic enzymes that hydrolyze lipids from the host cell to release free fatty acids, which are used as an energy source; (ii) the formation of building blocks for the synthesis of the bacterial cell wall, and (iii) modulation of the host immune responses (Rameshwaram et al., 2018). Our data showed that all the DEPs in the lipid metabolic pathway were down-regulated, which could help to explain the attenuated virulence and slow growth rate of the mutant strain Δ*mnmE*.

Deletion of *mnmE* down-regulated three core enzymes associated with arginine metabolism (ArcA, ArcB, and ArcC), which participate in the ADS. As a secondary metabolic pathway, ADS catalyzes the conversion of arginine to ornithine, as well as carbon dioxide and ammonia, with the concomitant release of adenosine triphosphate (Figure 4C). There are three main functions of the enzymes involved in the ADS: (i) production of energy via arginine metabolism, (ii) provision of carbamoyl phosphatase for the biosynthesis of citrulline or pyrimidines, and (iii) protection against acidic damage by the production of NH_3_. Based on the above theory, ADS involvement has been confirmed in the pathogenicity and adaptability to acidic conditions of many bacteria, such as *Streptococcus gordonii, Listeria monocytogenes, S. suis, Streptococcus pyogenes, Laribacter hongkongensis*, and *E*. *coli* (Liu et al., 2008; Smith et al., 2013; Fulde et al., 2014; Xiong et al., 2014; Sakanaka et al., 2015; Willenborg et al., 2016). A previous report has also stated that *mnmE* deficiency could affect the adaptability of *E*. *coli* to an acidic environment (Vivijs et al., 2016). This molecular mechanism can be explained by the down-regulated activity of the ADS through MnmE. Therefore, inhibition of the arginine metabolic pathway significantly contributed to reduced pathogenicity and arginine deiminase activity of the Δ*mnmE* mutant.

About two-thirds of the proteins (14 of 20) involved in the purine metabolic pathway were repressed in strain Δ*mnmE*. Previous studies have emphasized the importance of nucleotide biosynthesis in bacteria. Auxotroph mutants of nucleotide biosynthesis in *S. aureus, Salmonella*, and *S. pneumoniae* have already been associated with stunted growth (McFarland and Stocker, 1987; Polissi et al., 1998; Mei et al., 2003; Fitzsimmons et al., 2018). However, the biosynthesis of valine, leucine, and isoleucine, cofactor production, and vitamin metabolic pathways were activated, while the DEPs in these pathways were overlapped. The connection between the deletion of MnmE and activation of these pathways is unclear, thus further investigations are needed to determine exactly how MnmE regulates these pathway in *S. suis*.

In conclusion, our *in vitro* and *in vivo* studies clearly demonstrate the important roles of MnmE on the growth, pathogenicity, and arginine metabolism of *S*. *suis*. Consistently, TMT analysis also confirmed these results. These findings provide new insights to better understanding the function of MnmE as a central tRNA-modifying GTPase and stress the importance of this protein in the mRNA decoding process in bacterial pathogens.

## Data Availability

The datasets generated for this study can be found in ProteomeXchange Consortium, The mass spectrometry proteomics data have been deposited to the ProteomeXchange Consortium via the PRIDE partner repository with the dataset identifier PXD012716.

## Ethics Statement

All mice used in this study were purchased from the Wuhan Institute of Biological Products Co., Ltd. (Wuhan, China). The animal study protocol was approved by the Ethics Committee of Huazhong Agricultural University (Wuhan, China) and conducted in accordance with the Hubei Province Laboratory Animal Management Regulations of 2005. All efforts were made to minimize animal suffering.

## Author Contributions

RZ and YT conceived and designed this project. TG mainly performed the experiments. FY, WeL, and WaL performed some experiments. DZ, KY, ZD, QH, and RG analyzed the data. TG contributed to the development of the figures and tables. TG, RZ, and YT wrote the manuscript. All authors gave approval of the final version to be published and agreed to be accountable for all aspects of the work.

### Conflict of Interest Statement

The authors declare that the research was conducted in the absence of any commercial or financial relationships that could be construed as a potential conflict of interest.

## Supplementary Material

Figure S1 Transmission electron microscopy images of bacteria.

Table S1 Differentially expressed proteins in mutant strains.

## References

[B1] Agris P. F. (2008). Bringing order to translation: the contributions of transfer RNA anticodon-domain modifications. EMBO Rep 9, 629–635. 10.1038/embor.2008.104 18552770 PMC2475317

[B2] Bhat Z. S. Rather M. A. Maqbool M. Lah H. U. L. Yousuf S. K. Ahmad Z. (2017). Cell wall: a versatile fountain of drug targets in *Mycobacterium tuberculosis*. Biomed. Pharmacother. 95, 1520–1534. 10.1016/j.biopha.2017.09.036 28946393

[B3] Bohl T. E. Shi K. Lee J. K. Aihara H. (2018). Crystal structure of lipid A disaccharide synthase LpxB from *Escherichia coli*. Nat. Commun. 9:377. 10.1038/s41467-017-02712-9 29371662 PMC5785501

[B4] Courtney H. S. Dale J. B. Hasty D. I. (1996). Differential effects of the streptococcal fibronectin-binding protein, FBP54, on adhesion of group A streptococci to human buccal cells and HEp-2 tissue culture cells. Infect. Immun. 64, 2415–2419. 8698460 10.1128/iai.64.7.2415-2419.1996 PMC174091

[B5] de Greeff A. (2002). Contribution of fibronectin-binding protein to pathogenesis of *Streptococcus suis* serotype 2. Infect. Immun. 70, 1319–1325. 10.1128/IAI.70.3.1319-1325.2002 11854216 PMC127759

[B6] Decoster E. Vassal A. Faye G. (1993). MSS1, a nuclear-encoded mitochondrial GTPase involved in the expression of COX1 subunit of cytochrome c oxidase. J. Mol. Biol. 232, 79–88. 10.1006/jmbi.1993.1371 8392589

[B7] Degnan B. A. Fontaine M. C. Doebereiner A. H. Lee J. J. Mastroeni P. Dougan G.. (2000). Characterization of an isogenic mutant of *Streptococcus pyogenes* Manfredo lacking the ability to make streptococcal acid glycoprotein. Infect. Immun. 68, 2441–2448. 10.1128/IAI.68.5.2441-2448.2000 10768929 PMC97444

[B8] Eniyan K. Dharavath S. Vijayan R. Bajpai U. Gourinath S. (2018). Crystal structure of UDP-N-acetylglucosamine-enolpyruvate reductase (MurB) from Mycobacterium tuberculosis. Biochim. Biophys. Acta Proteins Proteom. 1866, 397–406. 10.1016/j.bbapap.2017.11.013 29203374

[B9] Feng Y. Zhang H. Wu Z. Wang S. Cao M. Hu D.. (2014). Streptococcus suis infection: an emerging/reemerging challenge of bacterial infectious diseases? Virulence 5, 477–497. 10.4161/viru.28595 24667807 PMC4063810

[B10] Ferrando M. L. van Baarlen P. Orru G. Piga R. Bongers R. S. Wels M.. (2014). Carbohydrate availability regulates virulence gene expression in Streptococcus suis. PLoS ONE 9:e89334. 10.1371/journal.pone.0089334 24642967 PMC3958366

[B11] Fislage M. Brosens E. Deyaert E. Spilotros A. Pardon E. Loris R.. (2014). SAXS analysis of the tRNA-modifying enzyme complex MnmE/MnmG reveals a novel interaction mode and GTP-induced oligomerization. Nucleic Acids Res. 42, 5978–5992. 10.1093/nar/gku213 24634441 PMC4027165

[B12] Fittipaldi N. Segura M. Grenier D. Gottschalk M. (2012). Virulence factors involved in the pathogenesis of the infection caused by the swine pathogen and zoonotic agent *Streptococcus suis*. Future Microbiol. 7, 259–279. 10.2217/fmb.11.149 22324994

[B13] Fitzsimmons L. F. Liu L. Kim J.-S. Jones-Carson J. Vázquez-Torres A. (2018). Salmonella reprograms nucleotide metabolism in its adaptation to nitrosative stress. mBio 9, e00211–00218. 10.1128/mBio.00211-18 29487237 PMC5829828

[B14] Frere J. M. Page M. G. (2014). Penicillin-binding proteins: evergreen drug targets. Curr. Opin. Pharmacol. 18, 112–119. 10.1016/j.coph.2014.09.012 25450065

[B15] Fulde M. Willenborg J. de Greeff A. Benga L. Smith H. E. Valentin-Weigand P.. (2011). ArgR is an essential local transcriptional regulator of the arcABC operon in *Streptococcus suis* and is crucial for biological fitness in an acidic environment. Microbiology 157(Pt 2), 572–582. 10.1099/mic.0.043067-0 20947575

[B16] Fulde M. Willenborg J. Huber C. Hitzmann A. Willms D. Seitz M.. (2014). The arginine-ornithine antiporter ArcD contributes to biological fitness of *Streptococcus suis*. Front Cell Infect. Microbiol. 4:107. 10.3389/fcimb.2014.00107 25161959 PMC4129364

[B17] Gao T. Tan M. Liu W. Zhang C. Zhang T. Zheng L.. (2016). GidA, a tRNA modification enzyme, contributes to the growth, and virulence of *Streptococcus suis* serotype 2. Front. Cell Infect. Microbiol. 6:44. 10.3389/fcimb.2016.00044 27148493 PMC4835480

[B18] Ge J. P. Catt D. M. Gregory R. L. (2004). *Streptococcus mutans* surface alpha-enolase binds salivary mucin MG2 and human plasminogen. Infect. Immun. 72, 6748–6752. 10.1128/IAI.72.11.6748-6752.2004 15501816 PMC523000

[B19] Ghazaei C. (2018). *Mycobacterium tuberculosis* and lipids: insights into molecular mechanisms from persistence to virulence. J. Res. Med. Sci. 23:63. 10.4103/jrms.JRMS_904_17 30181745 PMC6091133

[B20] Goyette-Desjardins G. Auger J.-P. Xu J. Segura M. Gottschalk M. (2014). *Streptococcus suis*, an important pig pathogen and emerging zoonotic agent—an update on the worldwide distribution based on serotyping and sequence typing. Emerg. Microb. Infect. 3:e45. 10.1038/emi.2014.45 26038745 PMC4078792

[B21] Gruening P. Fulde M. Valentin-Weigand P. Goethe R. (2006). Structure, regulation, and putative function of the arginine deiminase system of Streptococcus suis. J. Bacteriol. 188, 361–369. 10.1128/JB.188.2.361-369.2006 16385025 PMC1347268

[B22] Jing H. B. Yuan J. Wang J. Yuan Y. Zhu L. Liu X. K.. (2008). Proteome analysis of *Streptococcus suis* serotype 2. Proteomics 8, 333–349. 10.1002/pmic.200600930 18081191

[B23] Joh D. Wann E. R. Kreikemeyer B. Speziale P. Hook M. (1999). Role of fibronectin-binding MSCRAMMs in bacterial adherence and entry into mammalian cells. Matrix Biol. 18, 211–223. 10.1016/S0945-053X(99)00025-6 10429941

[B24] Li W. Liu L. Chen H. Zhou R. (2009). Identification of Streptococcus suis genes preferentially expressed under iron starvation by selective capture of transcribed sequences. FEMS Microbiol. Lett. 292, 123–133. 10.1111/j.1574-6968.2008.01476.x 19191874

[B25] Liu Y. Dong Y. Chen Y. Y. Burne R. A. (2008). Environmental and growth phase regulation of the *Streptococcus gordonii* arginine deiminase genes. Appl. Environ. Microbiol. 74, 5023–5030. 10.1128/AEM.00556-08 18552185 PMC2519279

[B26] Lun Z.-R. Wang Q.-P. Chen X.-G. Li A.-X. Zhu X.-Q. (2007). *Streptococcus suis*: an emerging zoonotic pathogen. Lancet Infect. Dis. 7, 201–209. 10.1016/S1473-3099(07)70001-4 17317601

[B27] Manickam N. Joshi K. Bhatt M. J. Farabaugh P. J. (2016). Effects of tRNA modification on translational accuracy depend on intrinsic codon–anticodon strength. Nucleic Acids Res. 44, 1871–1881. 10.1093/nar/gkv1506 26704976 PMC4770228

[B28] Margulieux K. R. Liebov B. K. Tirumala V. Singh A. Bushweller J. H. Nakamoto R. K.. (2017). *Bacillus anthracis* peptidoglycan integrity is disrupted by the chemokine CXCL10 through the FtsE/X Complex. Front. Microbiol. 8:740. 10.3389/fmicb.2017.00740 28496437 PMC5406473

[B29] Martinez-Vicente M. Yim L. Villarroya M. Mellado M. Perez-Paya E. Bjork G. R.. (2005). Effects of mutagenesis in the switch I region and conserved arginines of *Escherichia coli* MnmE protein, a GTPase involved in tRNA modification. J. Biol. Chem. 280, 30660–30670. 10.1074/jbc.M503223200 15983041

[B30] McFarland W. C. Stocker B. A. D. (1987). Effect of different purine auxotrophic mutations on mouse-virulence of a Vi-positive strain of Salmonella dublin and of two strains of Salmonella typhimurium. Microb. Pathogen. 3, 129–141. 10.1016/0882-4010(87)90071-4 2849016

[B31] Mei J.-M. Nourbakhsh F. Ford C. W. Holden D. W. (2003). Identification of *Staphylococcus aureus* virulence genes in a murine model of bacteraemia using signature-tagged mutagenesis. Mol. Microbiol. 26, 399–407. 10.1046/j.1365-2958.1997.5911966.x 9383163

[B32] Moukadiri I. Garzon M. J. Bjork G. R. Armengod M. E. (2014). The output of the tRNA modification pathways controlled by the *Escherichia coli* MnmEG and MnmC enzymes depends on the growth conditions and the tRNA species. Nucleic Acids Res. 42, 2602–2623. 10.1093/nar/gkt1228 24293650 PMC3936742

[B33] Nilsson K. Jäger G. Björk G. R. (2017). An unmodified wobble uridine in tRNAs specific for Glutamine, Lysine, and Glutamic acid from *Salmonella enterica* Serovar Typhimurium results in nonviability—Due to increased missense errors? PLoS ONE 12:e0175092. 10.1371/journal.pone.0175092 28430781 PMC5400242

[B34] Polissi A. Pontiggia A. Feger G. Altieri M. Mottl H. Ferrari L.. (1998). Large-Scale Identification of Virulence Genes from *Streptococcus pneumoniae*. Infect. Immun. 66, 5620–5629. 9826334 10.1128/iai.66.12.5620-5629.1998 PMC108710

[B35] Rameshwaram N. R. Singh P. Ghosh S. Mukhopadhyay S. (2018). Lipid metabolism and intracellular bacterial virulence: key to next-generation therapeutics. Future Microbiol. 13, 1301–1328. 10.2217/fmb-2018-0013 30256124

[B36] Rodionova I. A. Goodacre N. Do J. Hosseinnia A. Babu M. Uetz P.. (2018). The uridylyltransferase, GlnD, and tRNA modification GTPase, MnmE allosterically control *Escherichia coli* folylpoly-γ-glutamate synthase, FolC. J. Biol. Chem. 293, 15725–15732. 10.1074/jbc.RA118.004425 30089654 PMC6177579

[B37] Sakanaka A. Kuboniwa M. Takeuchi H. Hashino E. Amano A. (2015). Arginine-ornithine antiporter ArcD controls arginine metabolism and interspecies biofilm development of *Streptococcus gordonii*. J. Biol. Chem. 290, 21185–21198. 10.1074/jbc.M115.644401 26085091 PMC4571851

[B38] Sandberg A. Lindell G. Kallstrom B. N. Branca R. M. Danielsson K. G. Dahlberg M.. (2012). Tumor proteomics by multivariate analysis on individual pathway data for characterization of vulvar cancer phenotypes. Mol. Cell Proteomics 11:M112 016998. 10.1074/mcp.M112.016998 22499770 PMC3394958

[B39] Segura M. Fittipaldi N. Calzas C. Gottschalk M. (2017). Critical *Streptococcus suis* virulence factors: are they all really critical? Trends Microb. 25, 585–599. 10.1016/j.tim.2017.02.005 28274524

[B40] Sham L.-T. Jensen K. R. Bruce K. E. Winkler M. E. (2013). Involvement of FtsE ATPase and FtsX Extracellular Loops 1 and 2 in FtsEX-PcsB complex function in cell division of *Streptococcus pneumoniae* D39. mBio 4, e00431–e00413. 10.1128/mBio.00431-13 23860769 PMC3735124

[B41] Shi R. Villarroya M. Ruiz-Partida R. Li Y. Proteau A. Prado S.. (2009). Structure-function analysis of *Escherichia coli* MnmG (GidA), a highly conserved tRNA-modifying enzyme. J. Bacteriol. 191, 7614–7619. 10.1128/JB.00650-09 19801413 PMC2786596

[B42] Shippy D. C. Fadl A. A. (2014). tRNA modification enzymes GidA and MnmE: potential role in virulence of bacterial pathogens. Int. J. Mol. Sci. 15, 18267–18280. 10.3390/ijms151018267 25310651 PMC4227215

[B43] Smith J. L. Liu Y. Paoli G. C. (2013). How does *Listeria monocytogenes* combat acid conditions? Can. J. Microbiol. 59, 141–152. 10.1139/cjm-2012-0392 23540331

[B44] Szafran M. J. Kołodziej M. Skut P. Medapi B. Domagała A. Trojanowski D.. (2018). Amsacrine derivatives selectively inhibit mycobacterial topoisomerase I (TopA), impair *M. smegmatis* growth and disturb chromosome replication. Front. Microbiol. 9:1592. 10.3389/fmicb.2018.01592 30065714 PMC6056748

[B45] Takamatsu D. Osaki M. Sekizaki T. (2001). Thermosensitive suicide vectors for gene replacement in *Streptococcus suis*. Plasmid 46, 140–148. 10.1006/plas.2001.1532 11591139

[B46] Tan M.-F. Liu W.-Q. Zhang C.-Y. Gao T. Zheng L.-L. Qiu D.-X.. (2017). The involvement of MsmK in pathogenesis of the *Streptococcus suis* serotype 2. Microbiol. Open 6:e00433. 10.1002/mbo3.433 28102028 PMC5387306

[B47] Tan M. F. Gao T. Liu W. Q. Zhang C. Y. Yang X. Zhu J. W.. (2015). MsmK, an ATPase, contributes to utilization of multiple carbohydrates and host colonization of *Streptococcus suis*. PLoS ONE 10:e0130792. 10.1371/journal.pone.0130792 26222651 PMC4519317

[B48] Tang J. Wang C. Feng Y. Yang W. Song H. Chen Z.. (2006). Streptococcal toxic shock syndrome caused by *Streptococcus suis* serotype 2. PLoS Med. 3:e151. 10.1371/journal.pmed.0030151 16584289 PMC1434494

[B49] Trieu-Cuot P. Carlier C. Poyart-Salmeron C. Courvalin P. (1991). Shuttle vectors containing a multiple cloning site and a lacZα gene for conjugal transfer of DNA from *Escherichia coli* to Gram-positive bacteria. Gene 102, 99–104. 10.1016/0378-1119(91)90546-N 1864514

[B50] Umeda N. Suzuki T. Yukawa M. Ohya Y. Shindo H. Watanabe K.. (2005). Mitochondria-specific RNA-modifying enzymes responsible for the biosynthesis of the wobble base in Mitochondrial tRNAs: implications for the molecular pathogenesis of human mitochondrial diseases. J. Biol. Chem. 280, 1613–1624. 10.1074/jbc.M409306200 15509579

[B51] van de Rijn I. Kessler R. E. (1980). Growth characteristics of group A streptococci in a new chemically defined medium. Infect. Immun. 27, 444–448. 6991416 10.1128/iai.27.2.444-448.1980 PMC550785

[B52] Vivijs B. Aertsen A. Michiels C. W. (2016). Identification of genes required for growth of *Escherichia coli* MG1655 at moderately low pH. Front. Microbiol. 7:1672. 10.3389/fmicb.2016.01672 27826291 PMC5078493

[B53] Willenborg J. Koczula A. Fulde M. de Greeff A. Beineke A. Eisenreich W.. (2016). FlpS, the FNR-like protein of *Streptococcus suis* is an essential, oxygen-sensing activator of the arginine deiminase system. Pathogens 5:51. 10.3390/pathogens5030051 27455333 PMC5039431

[B54] Winterhoff N. Goethe R. Gruening P. Rohde M. Kalisz H. Smith H. E.. (2002). Identification and characterization of two temperature-induced surface-associated proteins of *Streptococcus suis* with high homologies to members of the arginine deiminase system of *Streptococcus pyogenes*. J. Bacteriol. 184, 6768–6776. 10.1128/JB.184.24.6768-6776.2002 12446626 PMC135470

[B55] Xiong L. Teng J. L. L. Watt R. M. Kan B. Lau S. K. P. Woo P. C. Y. (2014). Arginine deiminase pathway is far more important than urease for acid resistance and intracellular survival in Laribacter hongkongensis: a possible result of arc gene cassette duplication. BMC Microbiol. 14, 42–42. 10.1186/1471-2180-14-42 24533585 PMC3936950

[B56] Yim L. Martinez-Vicente M. Villarroya M. Aguado C. Knecht E. Armengod M. E. (2003). The GTPase activity and C-terminal cysteine of the *Escherichia coli* MnmE protein are essential for its tRNA modifying function. J. Biol. Chem. 278, 28378–28387. 10.1074/jbc.M301381200 12730230

[B57] Yim L. Moukadiri I. Bjork G. R. Armengod M. E. (2006). Further insights into the tRNA modification process controlled by proteins MnmE and GidA of *Escherichia coli*. Nucleic Acids Res. 34, 5892–5905. 10.1093/nar/gkl752 17062623 PMC1635325

[B58] Zhang C. Sun W. Tan M. Dong M. Liu W. Gao T.. (2017). The eukaryote-like serine/threonine kinase STK regulates the growth and metabolism of zoonotic *Streptococcus suis*. Front. Cell. Infect. Microbiol. 7:66. 10.3389/fcimb.2017.00066 28326294 PMC5339665

[B59] Zhong X. Zhang Y. Zhu Y. Dong W. Ma J. Pan Z.. (2018). The two-component signaling system VraSRss is critical for multidrug resistance and full virulence in *Streptococcus suis* serotype 2. Infect. Immun. 86:e00096–18. 10.1128/IAI.00096-18 29685990 PMC6013655

[B60] Zucchini L. Mercy C. Garcia P. S. Cluzel C. Gueguen-Chaignon V. Galisson F.. (2018). PASTA repeats of the protein kinase StkP interconnect cell constriction and separation of *Streptococcus pneumoniae*. Nat. Microbiol. 3, 197–209. 10.1038/s41564-017-0069-3 29203882

