# Supplementary figures and images for: MnmE, a Central tRNA-Modifying GTPase, Is Essential for the Growth, Pathogenicity, and Arginine Metabolism of Streptococcus suis Serotype 2
Source: Front Cell Infect Microbiol. 2019 May 24;9:173. doi: 10.3389/fcimb.2019.00173 (PMC6543552; doi:10.3389/fcimb.2019.00173)

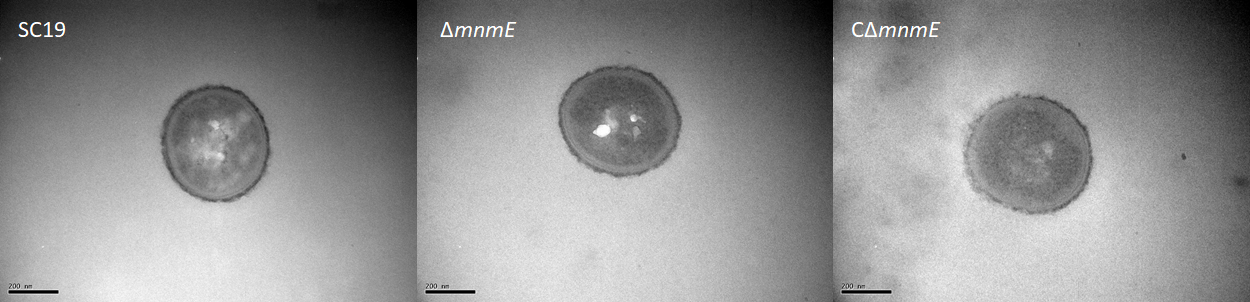

Supplement: Figure S1 — Transmission electron microscopy images of bacteria. [file Image_1.tif]
